# Is similarity in Major Histocompatibility Complex (MHC) associated with the incidence of retained fetal membranes in draft mares? A cross-sectional study

**DOI:** 10.1371/journal.pone.0237765

**Published:** 2020-08-17

**Authors:** Joanna Jaworska, Dawid Tobolski, Tomasz Janowski

**Affiliations:** 1 Department of Animal Reproduction with Clinic, Faculty of Veterinary Medicine, University of Warmia and Mazury, Olsztyn, Poland; 2 Department of Internal Diseases with Clinic, Faculty of Veterinary Medicine, University of Warmia and Mazury, Olsztyn, Poland; University College Dublin, School of Veterinary Medicine, IRELAND

## Abstract

The failure of the maternal immune system to recognize fetal antigens and vice versa due to MHC similarity between the foal and its dam might result in the lack of placental separation during parturition in mares. The aim of the study was to investigate the influence of MHC similarity between a mare and a foal on the incidence of retained fetal membranes (RFM) in post-partum mares. DNA was sampled from 43 draft mares and their foals. Mares which failed to expel fetal membranes within three hours after foal expulsion were considered the RFM group (n = 14) and mares that expelled fetal membranes during the above period were the control group (n = 29). Nine MHC microsatellites of MHC I and MHC II were amplified for all mares and foals. MHC compatibility and MHC genetic similarity between mares and their foals was determined based on MHC microsatellites. The inbreeding coefficient was also calculated for all horses. The incidence of RFM in the studied population was 33%. Compatibility in MHC I and MHC II did not increase the risk of RFM in the studied population of draft mares (P>0.05). Differences in MHC similarity at the genetic level were not observed between mare-foal pairs in RFM and control group (P>0.05). We suspect that RFM in draft mares may not be associated with MHC similarity between a foal and its dam. Despite the above, draft horses could be genetically predisposed to the disease.

## Introduction

The equine placenta is composed of maternal endometrial tissues and fetal allantochorionic tissues [1]. A partial or complete failure of the allantochorion to detach from the endometrium within 3 hours after foal delivery is a condition known as retained fetal membranes (RFM) and is frequent in post-partum mares [2, 3]. Interestingly, up to 54% of Friesian and heavy draft type mares suffer from RFM after parturition, and they appear to be more susceptible to RFM than other breeds [2–4]. Despite the above, the etiology of RFM has not been completely elucidated.

Parturition is often compared to a graft rejection-like reaction where the recognition of a foreign antigen triggers a characteristic outbreak of inflammatory processes [5]. At the end of pregnancy, the functioning of the maternal immune system changes, allowing for recognition of the fetal antigens expressed on fetal membranes [5, 6]. A recent study demonstrated that the fetal immune system, although immature, is also able to initiate an immune response against maternal antigens [7]. Both maternal and fetal antigens may be presented by the Major Histocompatibility Complex (MHC) [8]. Studies evaluating the influence of MHC similarity/dissimilarity on the outcome of transplantation in humans indicate that MHC I and MHC II similarity between the graft and host prolongs the survival of transplanted organs. In contrast, MHC I and MHC II dissimilarity significantly shortens their lifespan [9–11]. A comparison of the above process to parturition indicates that the expression of dissimilar MHC antigens on the maternal and fetal placenta can trigger a graft rejection-like reaction which is associated with the characteristic inflammatory outbreak during parturition that ends with the expulsion of fetal membranes [5, 6, 12]. However, if fetal and maternal antigens are similar, they may not be recognized as foreign by the respective immune systems, which can impair the inflammatory process during parturition [12].

Studies of Dutch Friesians indicate that a high level of inbreeding in foals could be responsible for the high incidence of RFM in this breed [13]. High inbreeding in a population generally increases the probability of two individuals having the same alleles of a gene or genes, such as MHC [14]. MHC I was also found to be expressed on the full-term equine placenta [15].

To the best of our knowledge, the association between maternal and fetal MHC and the incidence of RFM has never been studied in draft horses. We hypothesized that MHC similarity between a mare and a foal would increase the risk of RFM in draft mares. As indicated above, the only study investigating the possible genetic component of the RFM occurrence in mares was conducted by Sevinga et al. [13], and the effect of the inbreeding coefficient on RFM was determined. Hence, to be able to refer to this study, the inbreeding coefficient of foals and their dams suffering from RFM, and foals and their dams not affected by the disease was compared.

## Materials and methods

### Ethical note

Blood samples from mares and foals were taken during an annual parentage testing as required by studbook regulations. No experimentation was performed in view of European directive 2010/63/EU and the Polish laws related to ethics in animal experimentation. According to the European directive 2010/63/EU on the protection of animals used for scientific purposes chapter 1 article 1.5 “practices undertaken for the primary purpose of identification of an animal” do not need the approval of the Institutional Animal Care and Use Committee which was confirmed by the Ethics Committee for Animal Experimentation at the University of Warmia and Mazury in Olsztyn (LKE.065.07.2019). Owner of the animals informed consent and agreed on the use of blood samples.

### Animals

The study was performed on 43 clinically healthy draft mares aged 4–15 years and their newborn foals. All horses were bred in the same stud farm under identical housing and feeding conditions and with equal access to veterinary care. Pregnancies and deliveries were physiological and were monitored by a veterinarian. The failure to expel fetal membranes within 3 hours after foal expulsion and a necessity for veterinary intervention based on a veterinarian’s decision was regarded as RFM and treated. The mares were divided retrospectively in two groups: mares with RFM (N = 14) and control mares (N = 29).

### DNA isolation

Jugular venipuncture to 8.5 ml blood tubes with ACD Solution A of trisodium citrate, 22.0g / L; citric acid, 8.0 g / L; and dextrose 24.5 g / L, 1.5mL as anticoagulant was used for blood samples collection.

RBC Lysis Solution (Qiagen, Hilden, Germany, #158902) was used to isolate peripheral blood lymphocytes. 1 ml of the buffy coat was transferred to 3 ml of RBC Lysis Solution in a conical tube, then incubated for either 5 or 20 minutes for adult horses or foals, respectively. In the next step, the tube was centrifuged at 1500 rpm for 5 minutes, and the supernatant was removed. Pellet of lymphocytes was washed with PBS three times, and during the last wash, cells were transferred to a 1.5 ml Eppendorf tube. Supernatant was removed, and the lymphocytes were snap-frozen in liquid nitrogen. Next, collected lymphocytes were transferred to the ultra freezer to—80°C and stored in these conditions until DNA isolation.

DNeasy Blood & Tissue Kit (Qiagen, Hilden, Germany, #69506) was used to isolate genomic DNA according to the manufacturer’s instructions. Obtained DNA was stored at -20°C until further analysis.

### Microsatellites typing

Nine MHC microsatellites were amplified in 3 multiplex PCRs. Their distribution on the equine chromosome 20 is shown in S1 Fig. Primers’ sequences, fluorescent labels, amplicons’ length as well as which microsatellites were amplified in multiplex 1, 2 and 3 are given in Table 1. 2 μl of genomic DNA, 6.25μl of DreamTaq PCR Master Mix (2X) (Thermo Fisher Scientific, Waltham, Massachusetts, USA, #K1072). 0.2 μl of fluorescently labeled forward and reverse primers in a concentration 5 μM each and H_2_O_DD_ to a total volume of 14.5 μl was used per reaction well. PCRs were run in following conditions: 95°C for 3 min followed by 35 cycles of 95°C for 30 s, 60°C for 30 s, 72°C for 60 s and of 72°C for 10 min for the final extension. Electrophoresis on 3% agarose gel with ethidium bromide was performed to confirm the specificity of microsatellites’ amplification.

**Table 1:** Primers for nine MHC microsatellites, sizes of the amplicons and gene accession numbers and/or references where applicable.

	MICROSATELLITE	MHC Class region	PRIMER	SEQUENCE	Obtained product size	Label	Gene bank accession number/ reference
MULTIPLEX 1	**ABGe9030**	II	forward reverse	CCAGCAGACCTGCAAGAGTA AGCATGAGAGCCATGAAGGT	193–209	NED	FN414920.1
	**TKY3324**	II	forward reverse	AGCCGTCCTGTTCCAACTAA TGCCCCTTAAAACTCTGTCTTT	239–261	NED	AB217267.1
	**COR110**	I	forward reverse	TTTGGTCTTTGCAGGTATGG TCTCCCTTCCTCTTTGTTCC	197–214	FAM	EF531702
MULTIPLEX 2	**COR112**	II	forward reverse	TTACCTGGTTATTGGTTATTTGG TCACCCACTAAATCTCAAATCC	230–260	NED	[16]
	**TAMU30593**	I	forward reverse	GAAGCCCAGTCTGAGTGAAGAT AGATTTGGACCGAGAAAGTCTG	329–345	FAM	[17]
MULTIPLEX 3	**COR113**	II	forward reverse	TGTTTAGAACTCGCCAGGAG TCATCAGTTCCTTGCCTAGC	248–272	NED	[16]
	**COR114**	II	forward reverse	TCAAAATCCACACTCCCTTC TCCATAAAGAGTGGGACACTG	225–245	FAM	[16]
	**UM011**	II	forward reverse	TGAAAGTAGAAAGGGATGTGG TCTCAGAGCAGAAGTCCCTG	158–173	NED	AF195130
	**UMN-JH38**	I	forward reverse	TGTGTGTGCACCTGTCCTTT GATGGGAGGGAATGAGGAAT	149–157	FAM	EF531700.1

In the next step, 1μl of every PCR product was mixed with 14 μl of Hi-Di™ Formamide (Applied Biosystems^TM^, Foster City, California, USA, # 4311320) and 0.5 μl of GeneScan™ 500 LIZ™ dye Size Standard (Applied Biosystems^TM^, Foster City, California, USA, # 4322682) to final volume 15.5 μl on a 96-well plate. PCR products were then denatured at 95°C for 5 min and placed immediately on ice. DNA fragments were separated and sized on 3500xL Genetic Analyzer capillary sequencer (Applied Biosystems^TM^, Foster City, California, USA, # 4322682) and analyzed with GeneMapper TM 4.0 software (Applied Biosystems^TM^, Foster City, California, USA).

### Statistical analysis

#### Microsatellite analysis

Microsatellite loci were tested for deviations from the Hardy-Weinberg equilibrium and linkage disequilibrium with the use of GENEPOP v. 4.1 [18]. The frequencies of the null allele were analyzed using CERVUS v. 3.0 [19].

#### MHC compatibility

MHC compatibility was calculated for every mare and foal pair based on MHC microsatellite alleles. The analysis was performed separately for the microsatellites of MHC I and II class. The following categories of MHC compatibility were applied in this study (adapted from [12]):

- Mare compatibility (MC)–the MHC alleles of the foal are compatible with the MHC alleles of the mare, i.e., the foal does not have any MHC alleles that are not present in the mare. The mare's immune system does not recognize the foal’s MHC as foreign;
- Foal compatibility (FC)–the MHC alleles of the mare are compatible with the MHC alleles of the foal, i.e., the mare does not have any MHC alleles that are not present in the foal. The foal’s immune system does not recognize the mare’s MHC as foreign;
- Mare-foal compatibility (MFC)–the MHC alleles of the mare are compatible with the MHC alleles of the foal and vice versa. Neither the mare's or the foal's MHC is recognized as foreign by foal’s or mare’s immune system, respectively;
- No compatibility (NC)–both the mare’s and the foal’s MHC alleles are recognized as foreign by either the foal's or mare's immune system, respectively

If all loci in a given MHC class fell into an assigned compatibility category, the mare-foal pair was considered as compatible (MC/FC/MFC) in a given MHC class I, II). If one or more loci within an MHC class did not fulfill the assigned compatibility restrictions, the mare-foal pair was considered as incompatible (NC) in a given MHC class. The influence of MHC compatibility on the occurrence of RFM was analyzed in the R statistical package (R Development Core Team, 2013, http://www.R-project.org/). Logistic regression model was used with NC compatibility category as a referent.

#### MHC genetic similarity assessed by microsatellites

MHC genetic similarity was assessed by relatedness between the mare and the foal based on MHC microsatellites alleles obtained by microsatellite typing. The r_xy_ statistic, which is regarded as an unbiased estimator of relatedness between two individuals, was used [20, 21]. This estimator of relatedness accounts for the similarity in allele composition of two individuals by chance (identity by state; IBS) based on reference allele frequencies [20, 21]. We hypothesized that genetic similarity between mares and foals from the RFM group would be higher hence, they will be more genetically similar in MHC than between mares and foals from the control group. Calculations of r_xy_ were performed for every mare-foal pair based on obtained MHC I and MHC II alleles in Demerelate R package v. 0.9–3 [22] with the use of *r*_*xy*_ function.

Next, the values of r_xy_ were compared between RFM mares and their foals vs. the control group with the use of the Student’s t-test and presented as means ± SD.

#### Inbreeding coefficient

For the calculation of the inbreeding coefficient (IF), pedigree data obtained from the database of Polish Horse Breeders Association (https://baza.pzhk.pl/) was used. IF was calculated for every mare and foal in CFC software [23]. The results have not been normally distributed. For that reason, IF of mares and foals from the RFM and control group were compared with the Mann Whitney U test. Results were expressed by median values (interquartile range), separately for mares and foals.

Statistical analyses were performed in PS IMAGO 5, IBM SPSS Statistics v.25 statistical package (IBM Corporation, Armonk, NY, USA). The results were regarded as significant at P<0.05. The normality of data distribution was tested with the Shapiro-Wilk test.

## Results

### Incidence of RFM in mares

RFM occurred in 33% of post-partum mares in the studied population. One sample binomial proportion rate (Clopper-Pearson) for the proportion of RFM to the total number of mares equals 0.326 (95% CI; 0.191–0.485).

### Analysis of MHC microsatellites

Five loci showed significant departure from Hardy-Weinberg equilibrium, COR110 (P = 0.012), TAMU30593 (P = 0.0004), TKY3324 (P<0.0001), UMO11 (P = 0.013), ABGe9030 (P = 0.023). Linkage disequilibrium was detected in all loci pairs (P<0.05) except for UMN-JH38 and TKY3324; UMN-JH38 and COR112, UMN-JH38 and ABGe9030. Relatively high null allele frequencies were detected for loci COR110 (0.14) and TKY3324 (0.15). Based on the above results, all loci were retained in the further calculation.

MHC I and MHC II alleles of all mares and foals participating in the study are shown in the S1 Table.

### MHC compatibility and RFM

The mare-foal pairs in every MHC compatibility category are shown in Table 2. The logistic regression model demonstrated that none of the compatibility categories (MC, FC, MFC) in any MHC class (I, II) influenced the occurrence of RFM in the studied population of mares (Table 3). The odds ratio (OR) 2.25 was determined for MFC in MHC I (CI 95%; 0.36, 13.42). However, it was not significant (P>0.05).

**Table 2:** Compatibility between mare and foal pairs in a given MHC class: MHC class I, MHC class II in the retained fetal membranes (RFM) group and the control group of mares.

Mare-foal pairs	MHC class compatibility	MHC compatibility (%)			
		MC	FC	MFC	NC
RFM mare-foal pairs (n = 14)	MHC I	1 (7)	3 (21)	3 (21)	7 (50)
	MHC II	3 (21)	2 (14)	2 (14)	7 (50)
CONTROL mare-foal pairs (n = 29)	MHC I	3 (10)	5 (17)	4 (14)	17 (59)
	MHC II	6 (21)	3 (10)	4 (14)	16 (55)

MC–maternal compatibility; FC–foal compatibility; MFC–mare-foal compatibility

**Table 3:** Summary of the logistic regression model predicting the incidence of retained fetal membranes in mares.

**MHC Class**		**Tested variable**		**Estimate**^**a**^		**S.E.**^**b**^		**OR**^**c**^		**P-value**		**95% CI**^**d**^			
												**Lower bound**		**Upper bound**	
		Intercept		-1.04		0.53		0.35		0.05		0.12		1.00	
MHC I		MC + FC		0.34		0.80		1.40		0.67		0.29		6.71	
		MFC		0.97		0.94		2.65		0.30		0.42		16.73	
MHC II		MC + FC		0.16		0.75		1.18		0.83		0.27		5.09	
		MFC		-1.01		1.23		0.36		0.41		0.03		4.09	
**Model Summary**															
**Model**	**Deviance**	**AIC**^**e**^	**BIC**^**f**^	**df**^**g**^	**X**^**2**^		**P-value**		**McFadden R**^**2**^		**Nagelkerke R**^**2**^		**Tjur R**		**Cox & Snell R**^**2**^
H_0_	52.70	54.70	56.46	42											
H_1_	50.84	60.84	69.65	38	1.86		0.76		0.04		0.06		0.01		0.04

MHC I NC and MHC II NC were used as referents. NC–no compatibility; MC–mare compatibility; FC–foal compatibility; MFC–mare-foal compatibility

^a^ tested variable estimate

^b^ standard error of the parameter estimate

^c^ odds ratio

^d^ 95% confidence interval of the odds ratio

^e^ Akaike information criterion

^f^ Bayesian information criterion

^g^ degrees of freedom.

### MHC genetic similarity assessed by microsatellites and RFM

There were no differences in pairwise relatedness r_xy_ (t = 0.02, P>0.05) between RFM mare-foal pairs and the control group mare-foal pairs. Mean relatedness r_xy_ for RFM and control mare-foal pairs was identical at r_xy_ = 0.52 ± 0.25.

### Inbreeding coefficient

There was no difference in the IF of mares from the RFM and control group (U = 191, P>0.05) and foals from these groups (U = 189, P>0.05). Median IF of mares was 0.007 (0.01) and median IF of foals was 0.01 (0.03).

## Discussion

Our findings suggest that RFM in the studied population of draft mares may not be associated with MHC similarity assessed by microsatellites between the mare and the foal. Approximately one-third of the mares were affected by RFM, however, MC, FC, or MFC did not significantly influence the incidence of the disease. Moreover, the genetic similarity assessed by microsatellites of mare-foal pairs in the MHC region did not differ between RFM and control mares. In contrast, Benedictus et al. [12] found that compatibility between the calf and its dam (MFC in our study) increased the risk of retained fetal membranes in cows. These variations could be attributed to differences in methodology. Benedictus et al. [12] assigned individuals to known MHC haplotypes based on the alleles obtained from DNA sequencing. These haplotypes were used for further calculations. However, MHC microsatellites are commonly used to evaluate the equine MHC, including studies where MHC compatibility between horses is of importance [24–28]. Unlike DNA sequencing, microsatellites are only indirect markers of MHC, but despite the above, they are regarded as reliable indicators of MHC haplotype and MHC genes sequencing [16, 17, 24–32]. However, we acknowledge that microsatellite typing could be less effective than DNA sequencing in identifying possible differences and similarities in DNA.

Grunig et al. [33] observed no differences in the maternal leukocyte response to invading trophoblasts in MHC-compatible and incompatible pregnancies in mares. Invasive trophoblast cells are referred to as the chorionic girdle, and they can be detected from around day 25 of pregnancy. Around day 30 of pregnancy, chorionic girdle cells begin to express MHC I of fetal origin, which induces a leukocyte influx as part of the maternal immune response. The expression of MHC I decreases by day 45 [34]. In our study, parturition was normal in all studied mare-foal pairs, even when the mare and the foal were classified as MHC-compatible. In the control group, one mare had MFC in two MHC classes, and four mares had MFC in MHC class I. Despite the above, all control mares expelled fetal membranes physiologically. In the RFM group, five mares were incompatible in every MHC class, but they retained fetal membranes. The results reported by Grunig et al. [33] suggest that a maternal immune response could be induced even in an MHC-compatible pregnancy. Therefore it is possible that regardless of the applied method of MHC evaluation ([33], this study), there were still differences between MHC of the foals and its dam that could provoke an immune reaction.

Interestingly, the studied population was characterized by low IF values at around 0.01 for the foals and 0.007 for the mares, and no differences were noted between mares and foals from the experimental groups. In a study of Friesians, yet another breed that is highly susceptible to RFM, Sevinga [13] estimated IF values at 0.157 for foals and 0.145 for mares and reported a positive, but minor effect of IF on the incidence of RFM in that breed. The author suggested that the majority of Friesian mares and foals could be MHC-compatible due to the high values of IF. The IF of the draft and Friesian horses differs [13, 35, 36], but both breeds are more susceptible to RFM than others [2–4]. It should also be noted that the incidence of RFM in draft mares is similar across the studs in the country (personal communication to draft horse breeders). We speculate that other genetic factors shared by these breeds might play a role in RFM pathogenesis. In the cited study [13], the heritability estimates of RFM in Friesians ranged from 0.05 in mares to 0.1 in foals. A study of cows revealed that RFM could be heritable in this animal species [37]. A similar conclusion arises from research into the genetic background of placental retention in humans. Women born from pregnancies that terminated with a retained placenta or women whose partners were born from such pregnancies were at significantly higher risk of RFM [38]. The evidence from human transplantation medicine suggests that in addition to distinct factors, such as MHC mismatch between a donor and a host, differences in the non-MHC region, a minor histocompatibility complex, and specific MHC alleles could also lead to the rejection of the transplanted organ [39, 40]. The expulsion of the fetus and fetal membranes can be compared to transplant rejection [41, 42]; therefore, it can be speculated that the similarity of minor histocompatibility complex antigens and/or the presence of specific MHC alleles in mares and foals could influence the incidence of RFM.

MHC microsatellite typing is an indirect method of MHC evaluation [30]. However, MHC microsatellite typing is a well-established method employed in research studies investigating the role of MHC in the physiology and pathology of horses [27, 28], including the experiments where the immune response to foreign antigen is of major interest [24–26]. The analyzed microsatellites were found to accurately correspond to MHC haplotypes, namely the set of MHC alleles in an individual [16, 17, 29–32]. In this study, a comparison of MHC alleles in mares and foals demonstrated that MHC microsatellite typing is an effective technique. The applicability of MHC II microsatellites might be debatable because these molecules are not expressed in the equine placenta during pregnancy [43]. Nevertheless, the study investigates the parturition when the recruitment of various types of immune cells able to express MHC II has been reported in other species [44–48]. Unfortunately, immunological events that lead to and take place during parturition in horses remain unknown. Based on results reported in different species, it can be speculated that cells expressing MHC II, including lymphocytes and macrophages, can be present in mares during labor. Compatibility of the MHC II alleles between the donor and the recipient is routinely tested in human transplantation. It has been shown that match within HLA-DR (Human Leukocyte Antigen isotype DR, MHC II) between the donor and the recipient maybe even more important for the graft survival than match within HLA-A and–B (Human Leukocyte Antigen isotype A and B, MHC I) [9, 10, 49, 50]. Mechanism of the rejection of the transplanted organ is compared to immune reactions which take place during parturition. For that reason MHC similarity between the mother and the fetus is considered as a possible factor influencing decreased immune reaction during the retention of fetal membranes in cows [12]. The above and the confirmed influence of MHC II compatibility/incompatibility on the transplant success rates in humans [9, 10] indicate that the presented analysis of the associations between MHC II similarity and RFM was fully justified.

In conclusion, the incidence of RFM in draft mares may not be associated with MHC I and/or MHC II similarity between a foal and its dam. However, draft and Friesian mares appear to be more susceptible to RFM other breeds, which could suggest that genetic factors are involved in RFM pathogenesis.

## Supporting information

S1 Fig Map of used in the study intra-MHC I and MHC II microsatellites. MHC region is located on the equine chromosome 20.
(TIF)

S1 Table Alleles of the nine amplified MHC microsatellites loci. RFM–mares and their foals belonging to retained fetal membranes group (RFM); control–mares and their foals belonging to the control group.
(XLSX)

S2 Table Alleles of the nine amplified MHC microsatellites loci of the family trios (foal, dam, sire). RFM–foals with their dams and sires belonging to retained fetal membranes group (RFM); control–foals with their dams and sires belonging to the control group.
(XLSX)
