# Supplementary figures and images for: Is similarity in Major Histocompatibility Complex (MHC) associated with the incidence of retained fetal membranes in draft mares? A cross-sectional study
Source: PLoS One. 2020 Aug 17;15(8):e0237765. doi: 10.1371/journal.pone.0237765 (PMC7430710; doi:10.1371/journal.pone.0237765)

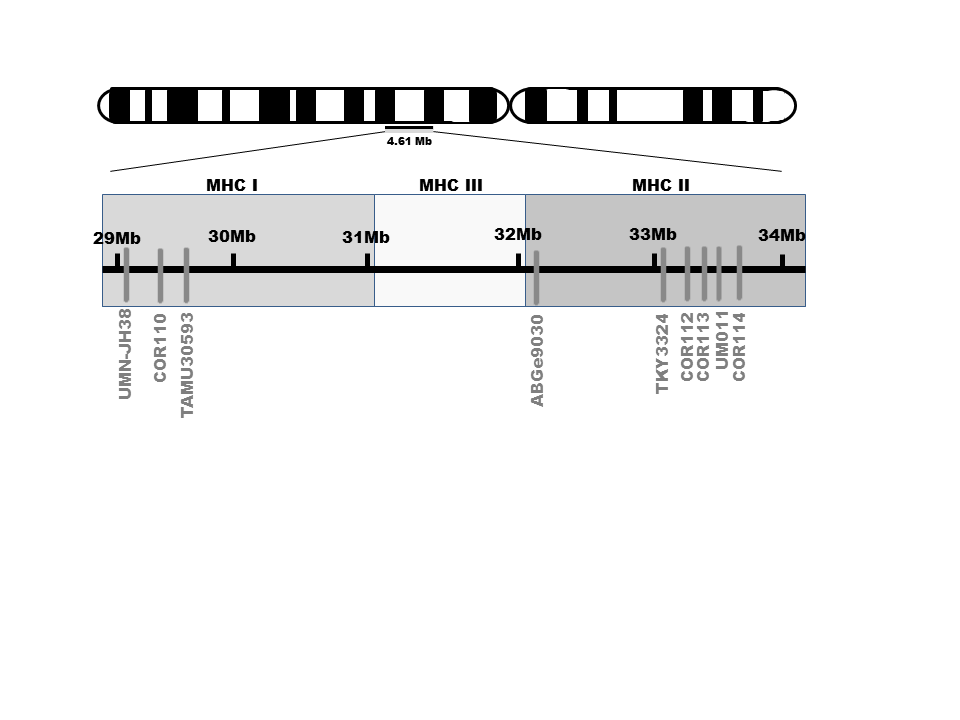

Supplement: S1 Fig — MHC region is located on the equine chromosome 20. (TIF) [file pone.0237765.s001.tif]
